# OA04 Muscle weakness in chronic rheumatic diseases: the need for early diagnosis and tailored exercise programme

**DOI:** 10.1093/rap/rkad070.004

**Published:** 2023-09-27

**Authors:** Thurkka Rajeswaran, Sarah Mackie

**Affiliations:** University of Leeds, Leeds, United Kingdom; Leeds Teaching Hospitals NHS Trust, Leeds, United Kingdom; University of Leeds, Leeds, United Kingdom; Leeds Teaching Hospitals NHS Trust, Leeds, United Kingdom

## Abstract

**Introduction:**

A 67-year-old female, diagnosed with giant cell arteritis (GCA) and polymyalgia rheumatica (PMR) in 2016, had remained on high doses of prednisolone since her relapse in 2020. Critical evaluation of her symptoms was needed to understand that her generalised pain was likely driven by muscle weakness, rather than PMR. Clear and honest communication was needed between the patient, her clinicians, and her physiotherapist, for all to feel safe to taper her prednisolone dose and adhere to a tailored high intensity rehabilitation regimen. Early diagnosis of muscle weakness was critical to preserve our patient’s mobility and quality of life.

**Case description:**

Our patient initially came under our care in 2016 when she presented with sore mouth, tongue claudication, knee and neck pain. Temporal artery biopsy confirmed GCA, and clinical assessment identified cooccurring PMR. She had an excellent initial response to glucocorticoid, with symptoms thereafter managed on subcutaneous methotrexate and low dose of prednisolone.

She suffered a considerable relapse in 2020 and was trialled on tocilizumab, leflunomide, sulfasalazine, all of which were not tolerated by the patient. She required high doses of prednisolone (18.5mg) alongside methotrexate (7.5mg) to manage her symptoms.

Three years on, she remained on high doses of steroids and methotrexate. Despite this, she suffered from generalised pain (across her knee, buttock, bilateral shoulder, upper arm, forearm and rib cage), that vastly limited her physical activity and quality of life. She had been an avid fell walker but had not been able to return to this activity due to her symptoms. During clinical review, it was evident that she suffered from proximal myopathy, needing to use her arms to rise from the chair, alongside weakness to her core muscles and rotators cuff.

This raised our suspicion that perhaps muscle weakness was mimicking her PMR symptoms. Muscle weakness that was likely driven by the long-term use of glucocorticoid. After lengthy discussion with our patient a management plan was agreed – her oral steroid dose would be tapered whilst simultaneously working with the physiotherapy team to rebuild her muscle strength.

Over the course of the next few months, we were able to effectively taper her prednisolone dose. She found her work with the physiotherapist extremely beneficial, reporting that she is now able to walk further and faster than before (albeit still with some pain). She has even recommenced fell walking – a feat that seemed almost impossible at the start of the year.

**Discussion:**

Our patient’s case highlights the need for clinicians to identify muscle weakness early. Whilst untreated or undertreated PMR may have an impact on muscle tissue through various influences (raised interstitial IL-6, alteration of muscle protein biosynthesis, myofascial inflammation) muscle weakness as seen here can occur subsequent to disuse atrophy or steroid myopathy. Muscle weakness can be quickly assessed in clinic by watching patient rise from their chair or walk down the corridor. Between appointments we can use ePROMs to monitor physical function; if gradual deterioration is seen, it is usually a red flag.

In our patient’s case, progress was only possible when all three parties involved in her care felt safe to proceed. As her clinicians we needed to know if it was safe to reduce her steroids, despite the worsening pain in the short term. Her physiotherapist needed to know it was safe to provide intensive support to increase exercise in a graded fashion. And most importantly, our patient needed to know it was safe to adhere to the plan, even though it was agonising and difficult. All three of these actions needed to occur simultaneously for the plan to be successful.

On trying to unpick what “feeling-safe” came down to, we felt it largely boiled down to effective communication between all parties involved, as well having the access to appropriate care. Our patient tells us she needed “open and honest conversations with clinicians you trust, clear information, a mutually agreed forward plan with agreed parameters regarding prednisolone reduction, and timely access when and if difficulties arose” to feel safe.

We ask the question to the audience on what they perceive “feeling-safe” means for them in decision making, and adhering to a plan despite set-backs?

**Key learning points:**

Our patient has learnt that “being able to trust the clinicians you are working with is critical to keeping going with the prednisolone reduction and physiotherapy, despite experiencing pain”. She stressed “as a patient never give up hope that you will be able to recover something that you feared lost.” Our learning echoes that of our patient. We emphasise the importance of honest and transparent communication, and the value of building a trusting relationship.

This case has helped us realise the importance of early diagnosis of muscle weakness and consideration of the root cause. We advocate for swift but effective assessment of muscle weakness in clinic, and the use of ePROMs in between clinics to monitor physical function.

We take away that “exercise” however is not a simple intervention and is only effective when it is embedded as a part of a holistic management plan. From this conference we hope to learn from colleagues how they have incorporated “exercise” into a comprehensive management plan, to create effective care for their patients.

